# The Effects of Oxidation on the Antithrombotic Properties of Tea Lipids against PAF, Thrombin, Collagen, and ADP

**DOI:** 10.3390/foods9040385

**Published:** 2020-03-26

**Authors:** Alexandros Tsoupras, Ronan Lordan, Jack Harrington, Rebecca Pienaar, Karen Devaney, Stephanie Heaney, Anastasios Koidis, Ioannis Zabetakis

**Affiliations:** 1 Department of Biological Sciences, University of Limerick, V94 T9PX Limerick, Ireland; Alexandros.Tsoupras@ul.ie (A.T.); ronan.lordan@ul.ie (R.L.); 15177688@studentmail.ul.ie (J.H.); 13197134@studentmail.ul.ie (R.P.); 15163334@studentmail.ul.ie (K.D.); 2 Health Research Institute, University of Limerick, V94 T9PX Limerick, Ireland; 3 Institute for Global Food Security, Queen’s University Belfast, BT9 5BN Belfast, Northern Ireland; sheaney16@qub.ac.uk (S.H.); t.koidis@qub.ac.uk (A.K.)

**Keywords:** tea, cardiovascular diseases, PAF, polar lipids, platelet aggregation, PUFA, MUFA

## Abstract

Tea provides health benefits, while oxidation is part of tea processing. The effect of oxidation on the antithrombotic properties of tea lipid extracts was evaluated for the first time. Total lipids (TL) extracted from fresh tea leaves and commercial tea powder, before and after 30–60 min of oxidation, were further fractionated into neutral lipids (NL) and polar lipids (PL). The antithrombotic bioactivities of tea TL, PL, and NL were assessed in human platelets against the inflammatory mediator platelet-activating factor. PL were further assessed against thrombin, collagen, and adenosine diphosphate, while their fatty acid composition was evaluated by GC-MS. PL exhibited the strongest antithrombotic effects against all platelet agonists and were rich in omega-3 polyunsaturated (ω3 PUFA) and monounsaturated (MUFA) fatty acids. A decline was observed in the antithrombotic activities, against all platelet agonists tested, for PL after 60 min of oxidation, and on their MUFA content, while their overall ω3 PUFA content and ω6/ω3 ratio remained unaffected. A synergistic effect between tea phenolic compounds and PL protects them against oxidation, which seems to be the rational for retaining the antithrombotic biofunctionalities of PL at a considerable favorable cardioprotective level, even after 60 min of tea oxidation. More studies are required to elucidate the mechanisms of the favorable synergism in tea PL extracts.

## 1. Introduction

Tea is brewed from the dried leaves of the plant *Camellia sinensis* and is one of the most widely consumed beverages in the world. Tea has many health benefits and tea is reported to contain nearly 4000 bioactive chemical compounds, including polar lipids (PL) and their subclasses of glycolipids, phospholipids, essential fatty acids (FA), and polyphenols such as catechins [1,2,3,4,5,6,7,8]. Tea can be mainly categorized into three types depending on the level of tea polyphenol oxidation. These include green tea (nonoxidized), oolong tea (partially oxidized), and black tea (fully oxidized) [1,2,3]. All types of tea are rich in phenolic compounds such as catechins (i.e., epigallocatechin gallate), flavonols (i.e., quercetin), phenolic acids (i.e., caffeic and gallic acids), and methylxanthines, containing approximately one-third the amount of caffeine compared with coffee [1,2,3]. Black tea also contains bioactive derivatives of catechins such as theaflavins and thearubigins, which are favourably formed at the expense of catechins during oxidation processing [1,2,3].

The health benefits of tea have previously been associated with putative antioxidant capabilities due to their phenolic contents that act against oxidative stress and its related unfavourable manifestations [2,3]. However, limited evidence exists that flavonoids can actually inhibit oxidative damage in vivo, since phenolic compounds have poor bioavailability and their antioxidant activities are affected and altered by their metabolic transformation. Indeed, inhibition of atherosclerosis in animal models was not associated with markers of change in oxidative damage by phenolic compounds [9]. The favourable effects of tea on several chronic disorders, including cardiovascular disease (CVD), seem to have little to do with the antioxidant properties of its phenolic compounds against oxidative stress, but more likely to do with other health related mechanisms, including the favourable effects of tea on endothelial function, inflammation, and the risk of thrombosis and platelet activation [2,3,10,11,12,13,14].

The beneficial effects of hot water extracts of tea against platelet aggregation and CVD have been studied since the early 1990s [8]. Several tea phenolic compounds have been identified as the active components that can inhibit platelet aggregation by affecting arachidonic acid (AA) and its eicosanoid-related pathways and through their effects against other platelet agonists and thrombo-inflammatory mediators, such as the platelet-activating factor (PAF), thrombin, collagen, and adenosine diphosphate (ADP) [2,3,10,11,12,13,14]. Tea catechins, isoprenyl gallates, theaflavin, and its galloyl esters in black tea extract are potent inhibitors of PAF synthesis and PAF-induced platelet aggregation [11,15]. Quercetin and gallic acid are also present in tea and have exhibited antiplatelet properties [12,13]. However, many of the observed antiplatelet effects of tea phenolic compounds in vitro are due to concentrations that cannot be attained in vivo [12,13,14], while consumption of large quantities of tea polyphenols may cause unwanted side effects [14].

Apart from its phenolic content, tea also contains other polar compounds, such as polar lipids (PL) (e.g., glycolipids and phospholipids), with several of them being rich in unsaturated FA, especially oleic acid (OA) and α-Linolenic acid (ALA), the latter being an essential omega-3 polyunsaturated FA (ω3 PUFA) [4,5,6,7,8]. OA, ALA, and other ω3 PUFA, such as the docosahexaenoic acid (DHA) and the eicosapentaenoic acid (EPA) have also exhibited antiplatelet effects of their own [16,17,18,19]. However, several PL of natural origin (e.g., from plants, animals, marine sources, microorganisms, food byproducts, etc.) and especially those bearing unsaturated FA possess much more potent antithrombotic properties against platelet aggregation and thus an overall protective effect against several chronic disorders including CVD [8,15,20,21,22,23,24,25,26,27,28,29].

Tea phenolic compounds along with tea PL and FA (e.g., OA and ALA) have also been found to be affected by seasonal variation and several manufacturing processes [1,4,5,6,7,8,30,31]. To our knowledge, both the antithrombotic activities of tea PL and the effect of oxidation on the biofunctionality and FA composition of tea PL compounds has not been reported to date. Therefore, the aim of this study was to evaluate the effects of oxidation levels on the biological activities of tea leaf lipids and PL for the first time. The antithrombotic properties of the tea lipids were assessed in human platelets against PAF, thrombin, ADP, and collagen. In addition, the changes on the FA composition of tea PL was also evaluated, in order to elucidate the effect of each level of oxidation on the structure activity relationships of tea derived bioactive PL.

## 2. Materials and Methods

### 2.1. Materials and Instrumentation

Analysis of platelet aggregation in human platelet-rich plasma (hPRP) was carried out on a Chronolog-490 two channel turbidimetric platelet aggregometer (Havertown, PA, USA), coupled to the accompanying AGGRO/LINK software package. 20G safety needles and evacuated sodium citrate S-monovettes for blood sampling were purchased from Sarstedt Ltd. (Wexford, Ireland). Platelet aggregation related consumables were purchased from Labmedics LLP (Abingdon on Thames, UK). Standard PAF, thrombin, BSA, and ginkgolide B were purchased from Sigma-Aldrich (Wicklow, Ireland), while collagen and ADP from Chronolog (Havertown, PA, USA) and aspirin (Bayer, Berlin, Germany) was purchased from a pharmacy (Bayer, Berlin, Germany). 1-hexadecyl-2-docosahexaenoyl-*sn*-glycero-3-phosphocholine standard (PCS) was purchased from Avanti Polar Lipids (Alabaster, AL, USA). Centrifugations were carried out on an Eppendorf 5702R centrifuge (Eppendorf Ltd., Stevenage, UK). Spectrophotometric analysis was carried out on a Shimadzu UV-1800 spectrophotometer (Kyoto, Japan) using a quartz 1 cm cuvette. All glass and plastic consumables, reagents, and solvents were of analytical grade and were purchased from Fisher Scientific Ltd. (Dublin, Ireland).

### 2.2. Samples of Tea Assessed before and after Oxidation Processing

Fresh tea leaves samples were obtained from a specific and widely available clone of the *Camellia senensis L.* plant, commonly known as black tea, withered for 16–18 h at room temperature, flash frozen with liquid nitrogen and grinded using a mortar and pestle. The leaves were left to defrost before they were exposed to different levels of oxidation, before (T0) and after 30 (T30) and 60 min (T60) of oxidation in the dark. Tea leaves were oxidized in an in-house oxidation unit developed for lab-scaled R&D tea processing experiments at Queen’s university, Belfast. The oxidation unit includes a shelf to hold the samples, a thermostat heating system to control temperature, and fans to ensure the flow of oxygen. When temperature stabilized at 32 °C and constant air stream (0.5 L/min), grinded tea samples (T30 and T60), were placed on the shelf within the oxidation unit. After 30 and 60 min, the leaves were removed and immediately placed in an oven at 103 °C to inhibit the oxidation reaction by drying. The freshly oxidized tea leaves were removed from the oven when they reached a constant weight at 103 °C. Dried leaves were milled to a powder using a ball mill for 5 min at a speed of 500 rpm. In addition, tea powder of commercial tea (CT) from a leading tea brand in Ireland (Barry’s Tea), was also analyzed as a control sample.

### 2.3. Extraction and Isolation of Total, Neutral, and Polar Lipids From Tea Leaves before (0 min) and after 30 and 60 min of Oxidation

Several tea samples of T0, T30, T60, and TC (*n* = 3 in each case), were homogenized mechanically by a Waring blender (Fisher Scientific Ltd., Dublin, Ireland) into a mixture of chloroform/methanol/water (1:2:0.8), the homogenized mixtures were filtrated, and their total lipids (TL) were extracted, as previously described [24,25], based on the Bligh and Dyer extraction method [32]. By the counter-current distribution method of Galanos and Kapoulas [33] all TL extracts were further fractionated into their neutral lipids (NL) and PL fractions, as previously described [24,25].

### 2.4. Human Platelet Aggregation Studies against PAF, Thrombin, Collagen, and ADP of Lipid Extracts from Tea Leaves before (0 min) and after 30 and 60 min of Oxidation

The evaluation of the antithrombotic properties of lipid extracts from tea leaves before (0 min) and after 30 and 60 min of oxidation against aggregation of human platelets induced by the inflammatory and thrombotic mediators, PAF and thrombin, and by the well-established platelet agonists collagen and ADP were performed in hPRP from healthy donors, as previously described [24,25,34]. The Ethics Committee of the University of Limerick approved the protocol, which was performed in accordance with the Declaration of Helsinki. Healthy donors were fully aware that their blood samples were used in our study and written consent was provided.

Briefly, the blood samples were collected from each donor by a phlebotomist in sodium citrate anticoagulant and were centrifuged at 194× *g* for 18 min at 24 °C with no brake applied. The supernatant hPRP was then transferred to polypropylene tubes at room temperature for the aggregation bioassays, whereas platelet-poor plasma (PPP) was obtained by further centrifuging the specimens at 1465× *g* for 20 min at 24 °C with no brake applied. hPRP was adjusted to 500,000 platelets/µL if required by addition of the respective volume of PPP according to the absorbance of the hPRP measured in spectrophotometer.

Standard stock solutions of active thrombin, collagen, and ADP dissolved in saline were further diluted in saline prior testing. Aspirin was also dissolved in saline prior testing. Aliquots of standard PAF stock solution in chloroform/methanol (1:1 *v*/*v*) were evaporated under a stream of nitrogen and redissolved in BSA (2.5 mg BSA/mL saline) to obtain PAF solutions with final concentrations into aggregometer cuvette ranging from 0.26 nM to 0.26 μM. The examined tea TL, NL, and PL samples and the standards of ginkgolide B and PCS, were also dissolved in BSA (2.5 mg BSA/mL saline).

Then, 250 µL of PRP was added to an aggregometer cuvette at 37 °C with stirring at 1000 rpm. The PRP was calibrated using the PPP as a blank. The maximum-reversible PAF, thrombin, collagen, and ADP-induced platelet aggregation was determined as 100% aggregation, which was also used as baseline (0% inhibition) in the absence of any sample, by adding appropriate amounts of each platelet agonist in the aggregometer cuvette, in order to reach specific final concentrations; for PAF approximately 0.1–1 nM, for thrombin approximately 0.01–0.4 U/mL, for collagen approximately 1–5 μg/mL, and for ADP approximately 2–10 μΜ.

Aggregation of hPRP induced by each agonist (PAF, thrombin, collagen, or ADP) was calculated first at 0% inhibition of baseline in a cuvette (100% aggregation) in the absence of any sample, whereas after the pre-incubation of hPRP with several amounts (μg) of the test samples for 2 min (a different cuvette was used for each amount of the sample tested), the same amount of the agonist was added and the reduced aggregation was calculated. Thus, a linear curve at the 20%–80% range of the percentage of inhibition against PAF, thrombin, collagen, and ADP-induced aggregation of hPRP to the concentrations of each sample was deduced. From this curve, the concentration (μg) of the test sample that led to 50% of the agonist induced aggregation of hPRP was calculated as the 50% inhibitory concentration value also known as the IC_50_ value (half-maximal inhibitory concentration) for each sample.

The resulting IC_50_ values were expressed as a mean value of the mass of lipid/standard (µg) in the aggregometer cuvette ± standard deviation (SD). All experiments were performed several times (*n* = 6) for each tea lipid sample (*n* = 6), using a different donors blood sample for each replicate.

### 2.5. Gas Chromatography-Mass Spectrometry of Polar Lipids from Tea Leaves before (0 min) and after 30 and 60 min of Oxidation

GC-MS analysis of the FA composition of the PL fractions from each tea sample (T0, T30, and T60) was carried out, as previously described [24,25].

### 2.6. Statistical Analysis

Normality for all IC_50_ values and FA composition obtained for each lipid sample was tested using Kolmogorov–Smirnov criterion. Subsequently, one-way analysis of variance (ANOVA) was used for all comparisons of IC_50_ values against PAF, thrombin, collagen, and ADP platelet aggregation, while Kruskal–Wallis nonparametric multiple comparison test was used for comparisons in the FA composition acquired from the CG-MS analysis. Differences were considered to be statistically significant when the *p*-value was less than 0.05 (*p* < 0.05). The data were analyzed using a statistical software package (IBM-SPSS statistics 25 for Windows, SPSS Inc., Chicago, IL, USA).

## 3. Results and Discussion

It is now recognized that the favorable effects of tea against several chronic disorders, including CVD, have more to do with its favorable effects on endothelial function, inflammation, and risk of thrombosis related to platelet activation and aggregation [2,3,10,11,12,13,14]. However, several manufacturing processes affect various compounds of tea leaves, such as their phenolic and other polar compounds like PL and FA (e.g., OA and ALA) [1,4,5,6,7,8,30,31].

In the present study, TL extracted from unoxidized and oxidized tea leaves (T0, T30, and T60) and from CT, were further fractionated into NL and PL fractions. Subsequently, their antithrombotic activities were assessed for the first time against aggregation of human platelets induced by the most well established platelet agonists and inflammatory mediators, namely PAF, thrombin, collagen, and ADP.

The obtained amounts of TL, PL, and NL (expressed as g of lipids per 100 g of tea sample) for all tea samples (T0, T30, T60, and CT) are given in Table 1. In these tea samples the main lipid components were found to be polar compounds, while the neutral lipids were found to be a minor component (Table 1).

The yields of PL extracts in all tea samples tested (approximately 7–10 g of PL per 100 g of tea sample) were at least two times higher than previously reported ones for tea (approximately 3–5 g of PL per 100 g of tea sample) [4,5]. The higher yield of polar compounds observed in all tea samples in the present study seem to be related with the different extraction methods employed than those used in the previously reported studies for tea polar lipids [4,5].

More specifically, in our study we used the well-established Bligh and Dyer extraction method for obtaining TL extracts [32], coupled with the efficient counter-current distribution of Galanos and Kapoulas [33], as previously described [24]. When this methodology was previously applied to other plant sources and related products that are also rich in polyphenols and bioactive polar compounds (e.g., red and white wine, musts, grapes, olive oil, sunflower oil, several kind of beers, and brewery byproducts), the bioactive phenolic compounds migrated to the PL fraction of the TL extracts within these conditions [25,26,27,28,35,36].

Similarly, the methodology applied in the present study facilitated the recovery of the majority of the tea polar compounds within the TL extracts of tea leaves, and from them the separation of the PL fractions that are rich in bioactive polar compounds and in which tea polyphenols comigrate. Therefore, in comparison with previous studies in tea PL [4,5], the higher amounts of PL extracted from the tea leaves in this study took place because of the comigration of phenolic compounds in these tea PL fractions due to the experimental extraction conditions applied. This is related to the fact that several phenolic compounds have similar to slightly higher polarity than classic PL subclasses of glycolipids and phospholipids. A characteristic example of that is the naturally occurring fatty esters of catechins in several tea varieties, namely phenolipids, which have a high antioxidant capacity and similar polarity and amphiphilic properties with classic PL subclasses, and thus comigrate into PL extracts [37,38].

**Figure 1:**
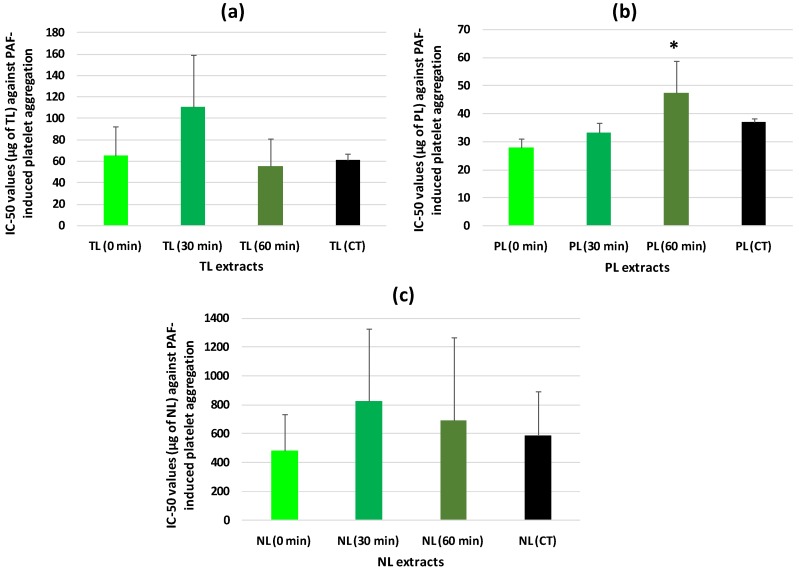
The antithrombotic effects of TL (**a**), PL (**b**), and NL (**c**) extracts from tea leaf samples, before (0 min) and after 30 and 60 min of oxidation, against PAF-induced aggregation of human platelets, in comparison to those of CT. Results are expressed as IC_50_ (half-maximal inhibitory concentration) values that reflect the inhibitory strength of each TL, PL, and NL extract against PAF-induced platelet aggregation and is expressed as mean values of μg of lipids in the aggregometer cuvette that causes 50% of inhibition on PAF-induced aggregation of platelets in hPRP ± SD. * Indicates statistical significant differences (*p* < 0.05). TL: Total lipids; PL: Polar lipids; NL: Neutral lipids; CT: Commercial tea samples; PAF: Platelet-activating factor; hPRP: Human platelet-rich plasma; SD: Standard deviation.

The in vitro antithrombotic properties of the TL, NL, and PL extracts from all tea samples, against PAF-induced aggregation of human platelets were expressed as IC_50_ values (Figure 1). The lower the IC_50_ value against PAF for a lipid extract the stronger its antithrombotic properties.

In this study, it was found for the first time that from all the lipid extracts tested that were derived from tea leaf samples at several stages of oxidation (T0, T30, and T60) and from CT, the PL extracts exhibited the strongest antithrombotic activities against PAF-induced aggregation of human platelets (Figure 1b).

It was also found that the TL extracts from all tea samples exhibited a potent anti-PAF effect (Figure 1a), which was slightly lower than that of their PL fractions. In contrast, all of the NL fractions had much lower anti-PAF effects (Figure 1c). This is also in accordance with previously reported outcomes for TL extracts from wine and beer that were also rich in higher amounts of PL, including bioactive phenolic and classic PL compounds. The TL also had higher bioactivities in comparison to the NL, which were less bioactive and in lower amounts [26,27,36].

The FA composition of the PL extracts from all tea samples are shown in Table 2. In all the tea PL samples, the PUFA and especially the essential ω3 PUFA ALA (18:3ω3), were the most abundant class of FA, followed by lower amounts of saturated fatty acids (SFA) such as the palmitic (16:0) and stearic (18:0) acids, and significantly less but considerable amounts of MUFA, such as OA (18:1c9). These results are in accordance with previous results for this tea variety [4,6,7,8,30,31,38], but also for other tea varieties [39,40,41].

In addition, in the majority of the tea PL, other MUFA were also present in less but considerable amounts, such as the palmitoleic (16:1c9), *cis*-vaccenic (18:1c11), and gadoleic (20:1c9) acids. Considerable amounts of ω6 PUFA were also present, with the most abundant being linoleic acid (LA; 18:2ω6), followed by much less but notable amounts of eicosatetraenoic acid (20:4ω6).

Interestingly, much less but considerable amounts of other long chain (LC) ω3 PUFA, such as the EPA (20:5ω3) and the DHA (22:6ω3), were also detected in these tea samples for the first time. It has been previously proposed that plant sources do not contain such LC-ω3 PUFA due to lack of appropriate enzyme machinery for producing them from ALA and LA, yet Guil et al. have reported the presence of low amounts of both EPA and DHA in several natural plants [41], which is in accordance with the results of this study.

PL from several natural sources and foods that are rich in such unsaturated FA, have exhibited potent bioavailability, biofunctionality, and antithrombotic properties, not only against PAF [20,21,22,23,24,25,26,27,28,29], but also against other platelet agonists such as thrombin [23,24,25,26,28], collagen, and ADP [25]. Therefore, the more potent anti-inflammatory and antithrombotic effects of such PL of natural origin, including plant-derived PL, against all these mediators and especially on PAF pathway and metabolism, have been translated to an overall favourable protective effect towards several inflammation-related disorders. Particularly CVD, in which PAF and these mediators are implicated [15,20,22]. The potent anti-PAF effects of the tea PL extracts that were rich in unsaturated FA, were of similar potency and efficacy to other anti-PAF PL extracts from natural sources that were also rich in ω3 PUFA and OA at their *sn*-2 position [23,24,25,26]. Consequently, the novel results in this study concerning the potent antithrombotic bioactivities of the tea PL characterized by their high unsaturated FA against PAF further supports the overall favorable health benefits of tea consumption.

Notably, the PL extracts from T0 exhibited significantly stronger anti-PAF effects in human platelets, than those of the PL extracts from T60 and CT (*p* < 0.05 in both comparisons). However, the PL extracts from T30 had an intermediate anti-PAF effect (Figure 1b). These results suggest that the more time applied in the oxidative process of the tea leaves the lower the bioactivities of their PL against PAF-induced human platelet aggregation.

**Figure 2:**
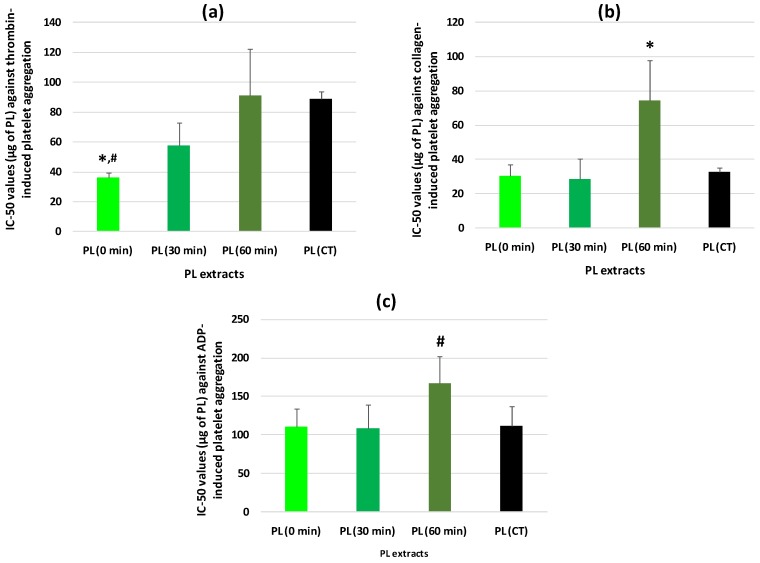
Antithrombotic effects of PL extracts from tea leaf samples, before (0 min) and after 30 and 60 min of oxidation, against thrombin (**a**), collagen (**b**), and ADP (**c**) induced aggregation of human platelets, in comparison to those of CT. Results are expressed as IC_50_ (half-maximal inhibitory concentration) values that reflect the inhibitory strength of each PL extract against thrombin, collagen, and ADP-induced platelet aggregation and is expressed as mean values of μg of PL in the aggregometer cuvette that causes 50% of inhibition on thrombin, collagen, and ADP-induced aggregation of platelets in hPRP ± SD. * Indicates statistical significant differences (*p* < 0.05); **#** indicates borderline statistical differences (0.05 < *p* < 0.10); PL: Polar lipids; CT: Commercial tea samples; ADP: Adenosine-5’-diphosphate; hPRP: Human platelet-rich plasma; SD: Standard deviation.

Since tea PL exhibited the most potent anti-PAF effects, in order to fully elucidate the overall effects of oxidation we further evaluated for the first time the antithrombotic properties of PL extracts from tea leaves at several oxidation levels (T0, T30, and T60) and from CT, against human platelet aggregation induced by thrombin, ADP, and collagen. The antithrombotic effects of these tea PL extracts against thrombin, collagen, and ADP were also expressed as IC_50_ values and are shown in Figure 2 (2a,b,c for each platelet agonist, respectively).

It was also found for the first time that PL extracts from all tea samples exhibited potent antithrombotic effects against thrombin, collagen, and ADP. Notably, it was also observed that the antithrombotic effects of these PL extracts tested against both thrombin and collagen were comparable and in the same order of magnitude to their relative biological effects against PAF, while their anti-ADP effects were less potent but also in considerable levels.

Furthermore, in a similar pattern to the results obtained against PAF, it was also found for the first time that the PL extracts from T0 had more potent anti-thrombin effects than the PL extracts from T60 and CT (*p* < 0.05 in both comparisons), while the PL extracts from T30 exhibited an intermediate anti-thrombin effect (Figure 2a). In the cases of collagen and ADP, the PL extracts from T60 had significantly less potent anti-collagen and anti-ADP effects than the PL extracts from all the other tea samples (Figure 2b,c) (*p* < 0.05 in all comparisons for collagen and borderline significance 0.05 < *p* < 0.10 in all comparisons for ADP). Again, these results for thrombin, collagen, and ADP, suggest that the more the time the oxidative process is applied to the tea leaves the lower the bioactivities of the PL against human platelet aggregation induced by these platelet agonists.

Nevertheless, the antithrombotic bioactivities of the PL extracts from all these tea samples against all platelet agonists (PAF, thrombin, collagen, and ADP) remained in the same order of magnitude for each agonist, and thus similar to the IC_50_ values obtained in human platelets for PL from other natural sources against PAF [23,24,25,26,27], thrombin [23,24,25,26,28], collagen, and ADP [25]. Consequently, although a decline was observed on the antithrombotic activities of the tea PL after 60 min of oxidation of the tea leaves, yet this reduction was not intense and as a result the antithrombotic properties of all tea PL in human platelets against all these platelet agonists were retained at substantial levels, comparable to those for PL from other natural sources [23,24,25,26,27].

In addition, the PL extracts from T0 exhibited similar anti-PAF bioactivities to their anti-thrombin and anti-collagen activity, while all these antithrombotic effects were stronger than their anti-ADP activity. Instead, the PL extracts from T30 and T60 exhibited significantly stronger anti-PAF effects than their anti-thrombin and anti-collagen effects, while all these antithrombotic activities were again stronger than their anti-ADP effects for these PL samples too. These results suggest that the aforementioned preservation of the antithrombotic activities of tea PL at substantial levels, even after 60 min of oxidation of the tea leaves, was more profound in the case of their anti-PAF effects, which further implies that the PL compounds with potent anti-PAF bioactivities that are present in the tea PL extracts are more sustainable against oxidation.

In addition, the in vitro antithrombotic properties of aspirin, ginkgolide B (a specific antagonist of PAF), and of PCS, a PAF-like molecule bearing ω3 PUFA (DHA) in its structure with both antagonistic and agonistic effects against the PAF-pathway were used as controls against aggregation of human platelets induced by PAF, thrombin, collagen, and ADP. The IC_50_ values of each of the controls are shown in Table 3. The platelet aggregation agonistic potency of PAF and of the PAF-like PCS molecule are also shown in the same table and are expressed as EC_50_ values. Again, the lower the EC_50_ value for a platelet agonist the higher its thrombotic potency.

Both the ginkgolide B and the PAF-like PCS molecule exhibited strong antithrombotic properties against all agonists of platelet aggregation, with higher specificity against the PAF-pathway. Furthermore, the amounts of the PCS needed for platelet aggregation were at least one order of magnitude higher than those needed for inhibiting platelet aggregation, while this agonistic effect on human platelets was found to be at least three orders of magnitude lower than that of PAF. Usually, acyl-phospholipids bearing ω3 PUFA at their *sn*-2 glycerol backbone, such as the tested PAF-like molecule, either possess a strong inhibitory or a weak agonistic effect or both effects (in different concentrations) against the PAF pathways of activating cells (including platelet aggregation). This is due to their structural resemblance to the PAF molecule, and thus their antagonistic effect for its receptor, but also due to the effect of DHA on the PAF-induced arachidonic acid pathway of eicosanoids [20]. Therefore, the dual agonistic and antagonistic effects of the PCS in different concentrations, further emphasizes its potent overall inhibitory effect against platelet aggregation with a specificity to the PAF-pathway.

It seems that tea PL possess stronger inhibitory effects against the PAF pathway than any possible agonistic effect from the constituent lipids in the PL fractions, and in this case, these components are acting as mostly PAF inhibitors. This result further emphasizes the high inhibitory potency of all tea PL extracts tested in this study that were comparable in the antiplatelet effects to several standard inhibitors of platelet aggregation such as aspirin and ginkgolide B. Furthermore, it was demonstrated using PCS that the PL themselves were involved in the antiplatelet response, not just the phenolic compounds (Table 3).

Moreover, the GC-MS analysis of the PL extracts from unoxidized (T0) and oxidized (T30 and T60) tea leaves revealed that the levels of ALA, their major ω3 PUFA, remained unaffected by this oxidation processing (Table 2). These results are in accordance with previously reported studies for this ω3 fatty acid. Therefore, even though a decline was observed in the levels of the lesser ω3 PUFA (reduction of EPA levels for both T30 and T60 and of DHA for T60), yet the overall ω3 PUFA content remained unaffected after oxidation of the tea leaves. Subsequently, the favorable low levels of the ω6/ω3 ratio observed in T0, which were lower than the value of 1/1 for this ratio, were retained in the same levels during oxidation (Table 2). These results further support the favourable ω3 PUFA content of the tea PL and thus their cardioprotective properties, even after 60 min of oxidation, since it has been proposed that low levels for the ω6/ω3 ratio has favorable effects in CVD and other chronic disorders [42].

At this point it is important to highlight that although the antioxidant tea polyphenols have little to do with a proposed in vivo protection against oxidative stress [9], they still play a crucial role as primary antioxidants against oxidation of lipids, including PL compounds, in several natural sources, foods, beverages, cosmetics, and lipid extracts [37,43,44]. In several leaves and cell membranes, a very substantial improvement in oxidative stability, bioavailability, and preservation of the bioactivities of both PL and polar phenolic compounds can be achieved by a copresence and synergism of both these polar compounds [37,43,44,45,46,47]. Subsequently, the presence of tea phenolic compounds in tea PL extracts, seem to facilitate the preservation of the bioactivities of the protected PL compounds. It is also possible that the previously reported oxidative stability of conjugative linolenic acids [48], may also contribute to the observed in this study preservation of ALA in tea PL after 60 min of oxidation.

On the other hand, in contrast to the ω3 PUFA, the overall levels of MUFA and those of individual OA (18:1c9), palmitoleic (16:1c9), *cis*-vaccenic (18:1c11), and gadoleic (20:1c9) acids were considerably reduced in the PL extracts of tea leaves that were oxidized for 60 min, a result that comes in accordance with previous studies in tea [31,49,50]. Especially the observed reduction in their major MUFA, OA, seem to be associated with the observed decline in the antithrombotic activities of these tea PL extracts against all platelet agonists tested, since OA has favorable antiplatelet effects [18,19]. It is also possible that the reduction of the antithrombotic activities of tea PL after 60 min of oxidation is related to an oxidation of some of the bioactive tea phenolic compounds that have previously reported antiplatelet effects, since they are labile to nonenzymatic oxidation during processing [37]. Nevertheless, more studies on the structure activity relationships are required in order to substantiate these effects.

## 4. Conclusions

It was found for the first time that tea PL extracts possess strong antithrombotic activities against the potent inflammatory mediator PAF, but also against other well-established platelet agonists, including thrombin, collagen, and ADP. The antithrombotic properties of tea PL were found to be comparable with classic antagonists and inhibitors of platelet aggregation for these agonists, such as aspirin, ginkgolide B, and PCS. Furthermore, the tea PL were also found to be rich in ω3 PUFA and MUFA, with a favorable ω6/ω3 ratio. However, a reduction of the antithrombotic activities of the tea PL extracts was observed after 60 min of oxidation, which was accompanied by a decline in their MUFA content. Nevertheless, these reductions were not intense, while the ALA and the overall ω3 PUFA content and the ω6/ω3 ratio of tea PL remained unaffected. Although the antithrombotic properties of all tea PL extracts declined due to oxidation, they remained in the same order of magnitude and comparable to those of PL from other natural sources. It is possible that the presence of phenolic compounds in these PL extracts may preserve their antithrombotic effects in considerable potency and protect them synergistically from the unfavourable effects of oxidation. However, more in vitro and in vivo studies are required in order to elucidate the preservation of the favourable effects of tea PL extracts due to the co-existing tea phenolic compounds and other bioactive PL subclasses. Finally, further studies are required on the putative utilization of highly antithrombotic tea-derived PL extracts, either for the fortification of functional foods or for the development of novel food supplements.

## Figures and Tables

**Table 1:** Yield of extraction of lipid content (TL, PL, and NL) of the tea samples before (0 min) and after 30 and 60 min of oxidation in comparison with commercial tea.

Tea Sample	TL *	NL *	PL *
**CT**	8.1 ± 1.5	0.8 ± 0.3	7.3 ± 1.2
**T(0)**	9.6 ± 4.9	1.6 ± 0.7	8.0 ± 5.6
**T(30)**	9.6 ± 0.6	0.3 ± 0.1	9.3 ± 0.3
**T(60)**	10.7 ± 4.0	0.9 ± 2.0	9.8 ± 2.0

* Expressed as mean values of g of lipids per 100 g of each marine source (mean ± SD, *n* = 6); TL: Total lipids; PL: Polar lipids; NL: Neutral lipids; CT: Commercial tea; T(0): Tea leaf samples before (0 min) oxidation; T(30): Tea leaf samples after 30 min of oxidation; T(60): Tea-leaf samples after 60 min of oxidation; SD: Standard deviation.

**Table 2:** The fatty acid profile of the PL extracts from tea leaf samples, before (0 min) and after 30 and 60 min of oxidation, in comparison to that of CT, expressed as a percentage of the total fatty acids of each sample (mean ± SD, *n* = 3).

Fatty Acid	0M	30M	60M	CT
**14:0**	0.700 ± 0.138 ^a^	0.135 ± 0.102 ^ab^	0.122 ± 0.003 ^b^	0.227 ± 0.019 ^ab^
**14:1**	ND	ND	ND	0.064 ± 0.013
**15:0**	0.120 ± 0.012 ^a^	0.047 ± 0.002 ^b^	ND	0.091 ± 0.003 ^ab^
**16:0**	24.60 ± 0.355 ^ab^	19.41 ± 1.117 ^a^	23.38 ± 0.947 ^ab^	28.11 ± 2.014 ^b^
**16:1 c9**	1.927 ± 0.033 ^ab^	1.461 ± 0.034 ^a^	1.752 ± 0.073 ^ab^	2.217 ± 0.080 ^b^
**17:0**	0.352 ± 0.010 ^b^	0.274 ± 0.017 ^ab^	0.020 ± 0.025 ^a^	0.332 ± 0.022 ^ab^
**18:0**	6.408 ± 0.241 ^ab^	8.496 ± 0.173 ^b^	7.930 ± 0.124 ^ab^	6.013 ± 0.099 ^a^
**18:1 c9**	9.965 ± 0.149 ^ab^	11.95 ± 0.274 ^b^	8.039 ± 0.114 ^ab^	7.840 ± 0.200 ^a^
**18:1 c11**	1.500 ± 0.115 ^b^	1.116 ± 0.048 ^ab^	0.787 ± 0.095 ^a^	1.184 ± 0.043 ^ab^
**18:2 c9, c12**	19.48 ± 0.222 ^a^	22.34 ± 0.262 ^a^	22.37 ± 0.247 ^a^	20.38 ± 0.839 ^a^
**18:3 c9, c12, c15**	29.78 ± 0.011 ^ab^	30.84 ± 0.529 ^ab^	34.22 ± 0.282 ^b^	27.44 ± 0.942 ^a^
**20:0**	0.343 ± 0.064 ^a^	0.442 ± 0.038 ^a^	ND	0.209 ± 0.032 ^a^
**20:1 c9**	1.105 ± 0.044 ^b^	0.698 ± 0.030 ^ab^	ND	0.379 ± 0.051 ^a^
**20:4 c5, c8, c11, c14**	0.344 ± 0.0109 ^ab^	0.397 ± 0.018 ^b^	ND	0.244 ± 0.033 ^a^
**20:5 c5, c8, c11, c14, c17**	0.732 ± 0.016 ^b^	0.655 ± 0.029 ^ab^	0.574 ± 0.050 ^ab^	0.339 ± 0.073 ^a^
**22:0**	1.052 ± 0.015 ^b^	ND	ND	0.319 ± 0.044 ^a^
**22:5 c7, c10, c13, c16, c19**	ND	ND	ND	0.375 ± 0.050
**22:6 c4, c7, c10, c13, c16, c19**	1.390 ± 0.113 ^b^	1.370 ± 0.155 ^ab^	0.518 ± 0.142 ^a^	1.122 ± 0.106 ^bc^
**ω3**	31.90 ± 0.215 ^ab^	32.86 ± 0.433 ^ab^	35.56 ± 0.176 ^b^	29.28 ± 1.060 ^a^
**ω6**	19.83 ± 0.228 ^a^	22.74 ± 0.271 ^b^	22.37 ± 0.247 ^ab^	20.62 ± 0.860 ^ab^
**ω6/ω3**	0.621 ± 0.011	0.692 ± 0.017	0.629 ± 0.010	0.704 ± 0.055
**SFA**	32.40 ± 0.532 ^ab^	28.80 ± 0.936 ^a^	31.64 ± 0.829 ^ab^	35.28 ± 1.905 ^b^
**MUFA**	15.55 ± 0.141 ^b^	15.28 ± 0.299 ^ab^	10.58 ± 0.051 ^a^	11.90 ± 0.287 ^ab^
**PUFA**	51.73 ± 0.413 ^ab^	55.60 ± 0.681 ^ab^	57.68 ± 0.805 ^b^	49.90 ± 1.873 ^a^

^a,b,c^ Mean values (*n* = 3), ± standard deviation, with different letters in the same row indicating statistical significant differences between the lipid compositions when mean are compared using Kruskal–Wallis nonparametric multiple comparison test (*p* ≤ 0.05). ω6/ω3 ratio uncertainty calculated using the following equation: ∆x/x = ([∆ω]6/ω6 + [∆ω]3/ω3) × ω6/ω3. Abbreviations: c: *Cis*; CT: Commercial tea; M: Minutes; MUFA: Monounsaturated fatty acids; PUFA: Polyunsaturated fatty acids; SFA: Saturated fatty acids; ND: Nondetectable.

**Table 3:** Antagonistic and agonistic effects of standards of aspirin, ginkgolide B, and 1-hexadecyl-2-docosahexaenoyl-*sn*-glycero-3-phosphocholine (PCS) against PAF, thrombin, collagen, and ADP on human platelets (hPRP).

	Inhibitory Effect *				Agonistic Effect **
Standard	PAF	Thrombin	Collagen	ADP	-
**Aspirin**	13.1 ± 5.0 (291.4 ± 111.8) #	3.5 ± 1.8 (77.4 ± 39.3)	3.0 ± 2.0 (66.4 ± 52.1)	3.6 ± 0.8 (79.6 ± 18.8)	ND
**Ginkgolide B**	5.5 ± 4.5 (65.4 ± 38.9)	9.2 ± 2.2 (86.6 ± 21.1)	12.3 ± 1.8 (116.0 ± 16.7)	ND	ND
**PCS**	0.3 ± 0.1 (1.5 ± 0.3)	0.6 ± 0.15 (3.1 ± 0.8)	0.5 ± 0.04 (2.3 ± 0.2)	1.5 ± 0.8 (7.4 ± 3.8)	17.6 ± 5.6
**PAF**	-	-	-	-	0.009 ± 0.001

* Results are expressed as IC_50_ (half-maximal inhibitory concentration) values that reflect the inhibitory strength of each standard against PAF, thrombin, collagen, ADP-induced platelet aggregation and is expressed as mean values of μg of standard in the aggregometer cuvette that causes 50% of inhibition on thrombin, collagen, and ADP-induced aggregation of platelets in hPRP ± SD. # In the parentheses the IC_50_ values for each standard are also expressed as μM concentration ± SD. ** Results are expressed as EC_50_ (half-maximal effective concentration) values that reflect the agonistic strength of each standard to induce aggregation of human platelets and is expressed as mean values of μM of standard in the aggregometer cuvette that causes 50% of aggregation of platelets in hPRP ± SD. ND: Not detected; SD: Standard deviation; PAF: Platelet activating factor; DHA: Docosahexaenoic acid; PC: Phosphatidylcholine.
